# Feeling Ostracized by Others’ Smartphone Use: The Effect of Phubbing on Fundamental Needs, Mood, and Trust

**DOI:** 10.3389/fpsyg.2022.883901

**Published:** 2022-07-01

**Authors:** Judith Knausenberger, Anna Giesen-Leuchter, Gerald Echterhoff

**Affiliations:** Department of Psychology, University of Münster, Münster, Germany

**Keywords:** phubbing, ostracism, fundamental needs, mood, interpersonal relations, trust game

## Abstract

With phubbing (i.e., “The act of snubbing someone… by looking at your phone instead of paying attention”) being a widespread phenomenon, a sound understanding of its emotional reverberations and consequences for interpersonal relationships is required. To the extent that phubbing is perceived as a momentary act of ostracism, it should influence both emotional and behavioral reactions. To address this issue empirically, we investigated effects of phubbing on variables previously shown to be affected by ostracism. Specifically, we examined in two studies how being phubbed affects participants’ mood, satisfaction of fundamental needs, feelings of being ostracized (Study 1 and 2) and trust (Study 2). In Study 1, participants remembered a situation in which they were either phubbed, phubbed someone else or experienced an attentive conversation. In Study 2 different phubbing behaviors were manipulated during an ongoing conversation. Results from both studies suggest that phubbing triggers negative mood and feelings of ostracism, and threatens fundamental needs. Study 2 revealed that these effects were stronger when phubbing occurred three times (vs. once). Study 2 further demonstrated behavioral consequences of phubbing, namely that trust in a trust game was reduced when participants were phubbed three times (vs. once). We discuss conceptual and practical implications of smartphone use for emotion regulation and interpersonal relations.

## Introduction

In 2021, there were 8.1 billion mobile phone subscriptions worldwide (Ericsson, 2021). By now, 97% of US Americans own a mobile phone (Pew Research Center, 2021) and use it quite frequently. Specifically, a study among college students revealed that this demographic uses their mobile phone for about 9 h a day (Roberts et al., 2014). This usage has many positive effects on relationships with friends and family members because it provides an easy way to stay connected (Leung and Wei, 2000; Salehan and Negahban, 2013). However, smartphone use can also have negative effects on social relationships. Using one’s mobile phone in the presence of others (i.e., phubbing, a portmanteau from the two words *phone* and *snubbing*; Klein, 2014; Stop Phubbing, n.d.) can make a bad impression on others and has been linked to reduced relationship quality (e.g., Roberts and David, 2016; Vanden Abeele et al., 2016; Beukeboom and Pollmann, 2021; Bröning and Wartberg, 2022).

Given the omnipresence of phubbing (Klein, 2014; Vanden Abeele et al., 2019), it is important to understand its harmful consequences. Therefore, we want to illuminate *how* phubbing can produce negative effects in interactions with physically present interaction partners. We argue that phubbing can be perceived as a momentary act of ostracism (i.e., ignoring and excluding others, Williams, 2007; also see Gonzales and Wu, 2016; Hales et al., 2018), thereby causing harm to the well-being of the co-present interaction partner (Williams, 2007). Phubbing represents a sudden diversion of attention away from the phubee, which conveys a lack of interest in the ongoing interaction with the phubee. We thus argue that phubbing can be perceived as ostracism because the phubber ignores the interaction partner (i.e., the phubbee, Chotpitayasunondh and Douglas, 2016) when using her or his mobile phone (Klein, 2014; Vanden Abeele et al., 2016; Stop Phubbing, n.d.). Moreover, the phubber potentially excludes the phubbee from an online interaction with another person who is addressed by the phubber. In these ways, phubbers are excluding and ignoring others, which matches the definitional criteria of ostracism.

Prior research has found ample evidence for negative consequences of ostracism for its targets (Williams, 2007). Specifically, ostracism induces social pain, threatens fundamental human needs, and causes negative mood. Thus, we hypothesize that phubbing causes negative mood and threatens fundamental human needs as well. Prior research has provided initial evidence for this assumption (Gonzales and Wu, 2016; Chotpitayasunondh and Douglas, 2018; Hales et al., 2018; Beukeboom and Pollmann, 2021; McDaniel and Wesselmann, 2021). The goal of our research was to replicate and extend this existing research (Study 1 and 2) in two important aspects. First, we extended previous research on consequences of phubbing by comparing the perspective of both phubbers and phubbees with a control condition (Study 1). With previous research on ostracism focusing mainly on targets of ostracism, a comparison of targets and sources of ostracism is highly needed (Zadro and Gonsalkorale, 2014). Exploring both perspectives of phubbing, that is, of both the phubbee and the phubber, enables a more comprehensive view of phubbing in its interactive context. Second, no research has yet investigated behavioral consequences of phubbing. Thus, we also explored whether the negative intrapersonal consequences of phubbing affect interpersonal behavior. Particularly, we predict that phubbing has similar effects on behavioral measures as ostracism (Study 2) and causes reduced behavioral trust shown toward phubbers.

### Phubbing as Ostracism

When engaging in phubbing, the phubber ignores the phubbee for a relatively short period (Vanden Abeele et al., 2016), and thus excludes him or her from a potential digital interaction with another person (Klein, 2014). Of course, mobile phones can be used for reasons other than interacting with someone else. However, mobile phones are frequently used for texting and other social media activities (Klein, 2014). Thus, the phubbee is likely to assume that the phubber engages in a remote interaction, thereby causing the phubbee to experience feelings of exclusion.

Previous research on ostracism has demonstrated that ostracism causes negative mood and threatens fundamental human needs of belongingness, self-esteem, control, and meaningful existence (i.e., need threat; e.g., Williams, 2007), but the intensity of these consequences depends on the temporal distance to the ostracism experience. In his temporal need-threat model, Williams (2009) distinguishes between a reflexive and reflective response to ostracism. The reflexive response is an immediate aversive experience, which is usually not affected by other variables (Hartgerink et al., 2015). Also, even minor cues of ostracism induce this reflexive threat to one’s needs and reduce positive mood (Spoor and Williams, 2007; Kerr and Levine, 2008; Giesen and Echterhoff, 2018). The reflexive response occurs because humans have an innate ostracism detection system that automatically detects all cues that might signal ostracism in the environment (Spoor and Williams, 2007; Kerr and Levine, 2008). When these cues are detected, the system alarms the individual by immediately causing negative mood and need threat. Since minor cues of ostracism are enough to activate the ostracism detection system, even short instances of being ignored and excluded, such as phubbing, should be enough to cause reflexive negative mood and need threat. Later, this reflexive response can be buffered by coping strategies and deliberate reflection on the experience (i.e., the reflective response). The initial reflexive response represents the immediate negativity of the experience, unaffected by coping mechanisms. Thus, in the present research we are interested in the effect of phubbing on reflexive need threats and mood. In a first exploration of this effect, Hales et al. (2018) demonstrated that remembering an instance of being phubbed causes feelings of ostracism, negative mood and need threat in phubbees.

Of course, the social pain response might be immediately buffered by the fact that the phubbers only ostracize the phubbees for a relatively short period of time. Specifically, they usually attend briefly to their mobile phone before re-focusing their attention on the real-life conversation, or they do so in between multiple phubbing episodes, where they divide their attention between their mobile phone and the concurrent conversation (Misra et al., 2016). Indeed, research on ostracism in which the ostracism experiences were followed by re-inclusion (Rudert et al., 2017) or in which people experienced partial ostracism (i.e., receiving one of two balls in an online ball-tossing game; Van Beest, 2016) caused less negative mood and lower need threat than did full-blown ostracism.

Most research on ostracism has investigated the consequences of being ostracized by a group (Williams et al., 2000; Williams and Jarvis, 2006; Wolf et al., 2014); only little research has manipulated ostracism by a single individual (e.g., Wirth et al., 2010). Arguably, being ostracized by one person might be less harmful than being ostracized by multiple individuals (Latané, 1981; DeWall et al., 2010). Still, the ostracism detection system should promptly react to the temporary ostracism by the phubber, causing at least some negative mood and need threat.

Given the high sensitivity of the ostracism detection system, prior research has demonstrated that ostracism is not merely painful for its direct targets but also for observers of ostracism (Wesselmann et al., 2009; Giesen and Echterhoff, 2018) and even the perpetrators (Legate et al., 2013). In their research, Legate et al. have shown that perpetrators of ostracism experience a threat to their autonomy and belongingness needs as well as negative mood. Also, more phubbing behavior has been found to be associated with more negative mood (Guazzini et al., 2021). Therefore, we expect phubbers to experience some need threat and negative mood, too.

Because ostracizers are responsible for the pain inflicted by ostracizing, it comes as no surprise that ostracized individuals have lower trust in the perpetrators (Twenge et al., 2007; Hillebrandt et al., 2011). Indeed, in prior research, ostracized individuals reported lower trust in others (Twenge et al., 2007) and showed lower behavioral trust in the so-called trust game (Hillebrandt et al., 2011). In this economic game (Berg et al., 1995), trust is operationalized by the amount of money or points a person (i.e., the sender) sends to an interaction partner (i.e., the receiver). This amount is multiplied, and the receiver can decide how much they want to give back to the sender. Thus, by initially sending money or points to the receiver, the sender can increase the profit of both parties. However, they are dependent on the decision by the receiver: The receiver could decide to send no money or points back, leaving the sender at a loss. Therefore, depending on how much the sender trusts the receiver to send back a reasonable amount of money or points, they will decide how much money or points they will initially send the receiver. By this process, the amount of money or points represents the level of the sender’s trust in the receiver. Importantly, ostracized individuals showed less behavioral trust toward the other player when that other player was their ostracizer (Hillebrandt et al., 2011). Thus, we expect that phubbed individuals, like ostracized individuals, demonstrate less behavioral trust toward their phubbers. Preliminary evidence for this assumption is provided by Przybylski and Weinstein (2013), who have shown that the mere presence of a smart phone reduces self-reported trust in the conversation partner. However, to our knowledge, no previous research has examined the consequences of phubbing on behavioral trust.

### Consequences of Phubbing

Prior research on the consequences of phubbing has revealed negative intra- and interpersonal consequences. In our research we aim to replicate and extend these findings. We further want to contribute to a deeper understanding of the negative consequences of phubbing by additionally investigating behavioral reactions of the phubbee toward the phubber. Prior research has solely focused their insights on self-reports by their participants. For example, people who had conversations in which a mobile phone was present or was used reported to experience less empathic concern (Misra et al., 2016), less interpersonal trust (Cameron and Webster, 2011), and reduced relationship (Roberts and David, 2016; Bröning and Wartberg, 2022) and friendship satisfaction (Sun and Samp, 2021). Overall, phubbing is perceived as inappropriate by phubbees, and the perceived inappropriateness increases with the frequency in which the mobile phone is used during the conversation (Klein, 2014). Some studies have also found positive associations between reported phubbing experiences and negative mood (do Nascimento Teixeira and de Assis Freire, 2020; Fellesson and Salomonson, 2020). However, most studies were correlational. To our knowledge, there are only a few published articles that have experimentally investigated the effect of mobile phone presence or usage on interactions with others who are physically present (Przybylski and Weinstein, 2013; Gonzales and Wu, 2016; Vanden Abeele et al., 2016; Hales et al., 2018; McDaniel and Wesselmann, 2021). For example, Przybylski and Weinstein (2013) demonstrated that the mere presence of a smart phone vs. a notebook during a dyadic conversation caused a reduction in perceived closeness, connection and conversation quality in both conversation partners. In another study, Vanden Abeele et al. (2016) showed that when someone interacts with a mobile phone during a conversation with another person, the phubbee perceives the phubber as being less attentive and polite.

However, the negative consequences of phubbing are not limited to the formation of interpersonal impressions and conversational quality, but they also cover effects like those of ostracism (Gonzales and Wu, 2016; Chotpitayasunondh and Douglas, 2018; Hales et al., 2018; McDaniel and Wesselmann, 2021). In fact, Hales et al. demonstrated that participants, who merely remembered being phubbed (vs. an attentive conversation vs. control) experienced feelings of ostracism, need threat and negative mood. Also, Gonzales and Wu (2016) as well as McDaniel and Wesselmann (2021) found that a manipulated phubbing episode during a face-to-face conversation caused feelings of exclusion in the participants. Chotpitayasunondh and Douglas (2018) found that imagining being phubbed increased negative mood, and decreased positive mood and feelings of belongingness. We aimed to replicate that phubbing causes negative mood, feelings of ostracism, and need threat for phubbees. Additionally, we examined negative effects of phubbing for phubbers and investigated a behavioral response to phubbing, that is, behavioral trust.

### Different Types of Phubbing

In the studies outlined above in which phubbing was manipulated in an ongoing interaction (Gonzales and Wu, 2016; Vanden Abeele et al., 2016), the study authors operationalized different key aspects of phubbing that can often be observed in the presence of others. Specifically, Vanden Abeele et al. manipulated the initiation type of the mobile phone interaction. In their first study, the confederate either used her or his mobile phone after the sound of a ringtone (i.e., reactive phubbing) or without the sound of a prior ringtone (i.e., proactive phubbing). Additionally, the authors altered the type of mobile phone interaction (reading a message in Study 1; writing a message in Study 2), as well as the frequency of mobile phone usage (i.e., 3x phubbing in Study 1; 1x phubbing in Study 2) across their experiments. Also, across their conditions, Gonzales and Wu implemented reactive and proactive mobile phone initiation as well as reading information and answering text messages. Furthermore, the mobile phone usage was announced by the confederate before they used it.

Even though these are all interesting aspects of phubbing, they were rarely manipulated separately in the same experiment. In fact, Vanden Abeele et al. (2016) only compared the consequences of proactive vs. reactive phubbing directly within one experiment, showing that proactive phubbing has more negative consequences than reactive phubbing, presumably due to a stronger violation of social norms. Similarly, Gonzales and Wu (2016) merely compared reading vs. writing in their experiment. They found that compared to being phubbed by a person who is reading information, being phubbed by someone who is writing messages to another person did not affect need threat and mood of the participants. Thus, some interesting aspects of phubbing were combined within the same condition or were only varied *between* different experiments (e.g., frequency of phubbing). Thus, the specific influence of each individual aspect could not be identified. From our view, an experimental comparison of these different aspects of phubbing provides important insights into the circumstances under which phubbing negatively affects interpersonal relationships. Therefore, we manipulated different aspects of phubbing behavior within one experiment and investigated their effect on the phubbee’s fundamental human needs, mood, and behavioral trust toward the phubber.

### Goals of the Present Research

We conducted the present research to experimentally investigate the consequences of phubbing on the phubbee’s well-being and behavioral responses toward the phubber. Prior research has demonstrated that phubbing causes feelings of being ostracized (Gonzales and Wu, 2016; Hales et al., 2018; McDaniel and Wesselmann, 2021) as well as negative mood and need threat in the phubbee (Hales et al., 2018). Our present research replicates and extends these prior studies by investigating the effect of phubbing on the reflexive satisfaction of fundamental human needs, mood, and feelings of ostracism (Studies 1 and 2). Specifically, we investigate for the first time how these negative intrapersonal consequences of phubbing translate into behavior and, therefore, we assessed behavioral trust (Berg et al., 1995) toward the phubber (Study 2).

In Study 1, participants were asked to remember a situation in which they were phubbed by another person, in which they have phubbed another person or in which they were having an attentive conversation. In Study 2, we manipulated phubbing in a face-to-face conversation between a confederate and participant and additionally varied the type (reactive vs. proactive, writing vs. reading) as well as the frequency (once vs. three times) of phubbing.

Building on prior ostracism research (Williams, 2007; Legate et al., 2013), we hypothesized that phubbing causes reflexive social pain in both the phubbee and the phubber, as indicated by negative mood, need threat, and feelings of ostracism. Because the ostracism detection system always alarms the individual when any cue of ostracism is detected (Spoor and Williams, 2007; Kerr and Levine, 2008), we expect phubbing to have a negative effect on need threat, mood and feelings of ostracism.

Specifically, we predict that the phubbee will show reduced trust toward the phubber. Concerning the different types of phubbing, we predict that reactive phubbing might be perceived as more acceptable than proactive phubbing because individuals might regard answering a received message as a social obligation and expect others to do so (Vanden Abeele et al., 2016). Reading a message might also have fewer negative consequences than typing an answer because the latter might require an even stronger focus on the mobile phone (Vanden Abeele et al., 2016). Finally, as pointed out by Klein (2014), the more often phubbing occurs, the more negative it might be. Thus, phubbing someone three times is predicted to have more negative effects than phubbing someone once.

## Study 1

Given the similarities between phubbing and ostracism, we expect that phubbing causes the phubbee to experience a threat of their fundamental needs of belongingness, self-esteem, control, and meaningful existence, as well as negative mood and feelings of ostracism (also see Hales et al., 2018). In addition, since even perpetrators of ostracism experience negative consequences of their behavior (Legate et al., 2013), we also expect phubbers to experience reduced need satisfaction and negative mood. As pointed out by Giesen and Echterhoff (2018), the ostracism detection system might mainly warn the individual by inducing negative mood because of its high informational value (Schwarz and Clore, 1983, 1996). Therefore, we specifically expect that while phubbees and phubbers might not differ in the extent of their negative moods, phubbers might experience less need threat than phubbees. To test our prediction, we conducted an online experiment, in which participants were asked to either remember and describe a situation in which they have experienced phubbing, in which they have phubbed someone else, or in which they had an attentive conversation with another person.

### Methods

#### Participants and Design

Data of this study are part of a larger exploratory online survey on phubbing behavior. To determine the required sample size, we calculated a power analysis with G*Power (Faul et al., 2009) based on the comparison between the Phubbee condition and the Attentive Conversation condition, with *d* = 0.64 (Hales et al., 2018), 1-β = 0.95, and α = 0.05. The analysis revealed a required sample size of *n* = 108 participants for these two conditions, resulting in a required sample size of *N* = 162 for all three conditions. To be able to detect a potentially smaller effect size for the Phubber condition, we recruited an additional 10% of participants (i.e., *N* = 179).In total, 182 participants answered this survey. Part of this survey involved having participants remember a situation in which they experienced being phubbed by another person (Phubbee condition), in which they had phubbed another person (Phubber condition), or in which they were having a conversation with an attentive other (Attentive Conversation condition). Participants were randomly assigned to these conditions. Five participants in the Attentive Conversation condition reported that the other person used their mobile phone during the conversation (i.e., has phubbed the participant). Thus, those five participants were excluded from further analyses. Another participant in the Attentive Conversation condition was excluded from further analyses because she or he did not describe a conversation but instead described a different situation. Additionally, four participants in the Phubber condition were excluded since they described a situation in which they were the phubbee instead of the phubber. Finally, one participant was excluded from further analyses because they did not describe any situation at all. The remaining sample size consisted of *N* = 170 participants (144 female, 26 male) with a mean age of 29.26 years (*SD* = 9.86 years).

#### Procedure

Upon opening the link to the online survey, participants received information about the study and agreed to participate by clicking a corresponding button. They were informed that they could withdraw from participating at any time during the study by simply closing the browser window. Data from these participants were not included in our analyses.

After having agreed to participate, the Phubbee, Phubber and Attentive Conversation conditions were manipulated modifying the essay manipulation of ostracism (Pfundmair et al., 2015). Thereafter, need satisfaction, feelings of being ostracized and mood were assessed. Participants in the Attentive Conversation condition were further asked whether their conversation partner had used her or his mobile phone during the conversation (response options: yes, no, I don’t know). If they answered ‘‘yes,’’ they were excluded from further analyses^1^. Afterward, potential moderators^2^ and demographic data (i.e., gender, age, educational level, occupation) were assessed. At the end of the study, participants were debriefed and received the opportunity to participate in a lottery of online store vouchers as compensation for their participation (we raffled four vouchers worth 20 € and four vouchers worth 10 €).

#### Materials

##### Essay Manipulation

We adapted the essay manipulation by Pfundmair et al. (2015) to test the present hypotheses. Participants in the Phubbee condition were instructed to remember a past conversation in which they were phubbed by their conversation partner. They were asked to remember a situation in which only they and one other person were involved and to write a detailed description of this situation, to describe their feelings, thoughts, and behavior. Participants in the Phubber condition received the same instruction, except they were asked to remember a situation in which they had phubbed someone else. Participants in the Attentive Conversation condition were asked to remember a conversation with one other person in which this person gave them her or his full attention. Before these instructions, participants in the Phubbee and Phubber conditions received a definition of “phubbing” (i.e., “phubbing describes the behavior of a person who uses her or his mobile phone during a conversation instead of focusing her or his attention on her or his conversation partner”).

##### Need Satisfaction

Need satisfaction was assessed by 20 items concerning the need to belong, need for self-esteem, need for control, and need for meaningful existence. For this purpose, we adapted the need-threat scale by Williams (2009) to fit the present context (e.g., “I felt like an outsider”, α = 0.95). Participants were asked to indicate on 5-point Likert scales (1 = *not at all*; 5 = *very*) how they felt while they were being phubbed.

##### Mood

To assess the mood of the participants during the conversation, they were asked to indicate to what extent they felt each of 28 emotional states on 5-point Likert scales (1 = *not at all*, 5 = *very*; e.g., angry, proud, nervous; Williams, 2009; PANAS, Krohne et al., 1996; α = 0.94). Higher values indicate a more positive mood.

##### Feelings of Ostracism

Two items measured feelings of ostracism (e.g., “I was excluded”; Williams, 2009; *r*_*SB*_ = 0.92). Again, participants indicated how they felt during the conversation on 5-point Likert scales (1 = *not at all*; 5 = *very*).

### Results

#### Needs

A one-factorial ANOVA with the conditions Phubbee, Phubber, and Attentive Conversation and need satisfaction as the dependent variable revealed a significant effect of the condition, *F*(2, 167) = 86.90, *p* < 0.001, η*_*p*_*^2^ = 0.51, 90% CI = [0.42;0.57]. Multiple comparisons, using the Bonferroni correction (Holm, 1979), revealed significant differences between all three conditions (see Table 1 for inferential statistics). Confirming our hypotheses, participants in the Phubbee condition reported the least need satisfaction (*M* = 2.48, *SD* = 0.69), followed by participants in the Phubber condition (*M* = 3.00, *SD* = 0.70). Participants in the Attentive Conversation condition reported most need satisfaction (*M* = 4.11, *SD* = 0.59). Thus, phubbing was related to need threat in both phubbees and phubbers.

**TABLE 1:** Phubbing effects on reported need satisfaction: multiple comparisons between experimental conditions.

	Need satisfaction				
	
	Δ *M*	*SE*	*P*	*d*	95% CI
Phubbee vs. Phubber	−0.52	0.12	<0.001	0.76	[0.38; 1.13]
Phubbee vs. Attentive communication	−1.64	0.13	<0.001	2.56	[2.05; 3.07]
Phubber vs. Attentive communication	−1.12	0.13	<0.001	1.72	[1.30; 2.15]

*n (Phubbee) = 54, n (Phubber) = 62, n (Attentive Conversation) = 54.*

#### Mood

Another ANOVA was conducted to test the effect of our three conditions on mood. Again, there was a significant effect of the condition, *F*(2, 167) = 46.98, *p* < 0.001, η*_*p*_*^2^ = 0.36, 90% CI = [0.26; 0.44]. Multiple comparisons revealed that phubbees (*M* = 2.82, *SD* = 0.53) and phubbers (*M* = 3.04, *SD* = 0.61) did not significantly differ from each other in their mood (see Table 2 for the inferential statistics). Those who wrote about an attentive conversation experienced significantly more positive mood (*M* = 3.93, *SD* = 0.75) than did participants in both other conditions. Again, these results show that phubbing has negative consequences for both phubbees and phubbers.

**TABLE 2:** Phubbing effects on reported mood: multiple comparisons between experimental conditions.

	Mood				
	
	Δ *M*	*SE*	*p*	*d*	95% *CI*
Phubbee vs. Phubber	−0.22	0.12	0.207	0.38	[0.007; 0.74]
Phubbee vs. Attentive communication	−1.11	0.12	<0.001	1.72	[1.27; 2.16]
Phubber vs. Attentive communication	−0.90	0.12	<0.001	1.55	[1.14; 1.97]

*n (Phubbee) = 54, n (Phubber) = 62, n (Attentive Conversation) = 54.*

#### Feelings of Ostracism

The third ANOVA with feelings of being ignored and excluded also revealed a significant effect of our conditions, *F*(2, 167) = 116.41, *p* < 0.001, η*_*p*_*^2^ = 0.58, 90% CI = [0.50;0.64]. Multiple comparisons showed that all three conditions differed significantly from each other (see Table 3 for the inferential statistics; Phubbee: *M* = 3.51, *SD* = 1.02; Phubber: *M* = 1.98, *SD* = 0.99; Attentive Conversation: *M* = 1.07, *SD* = 0.23). Thus, both phubbees and phubbers felt ostracized.

**TABLE 3:** Phubbing effects on reported feelings of being ignored and excluded multiple comparisons between experimental conditions.

	Feeling ignored and excluded				
	
	Δ *M*	*SE*	*P*	*d*	95% CI
Phubbee vs. Phubber	1.53	0.16	<0.001	1.53	[1.11; 1.94]
Phubbee vs. Attentive communication	2.44	0.16	<0.001	3.31	[2.73; 3.89]
Phubber vs. Attentive communication	0.90	0.16	<0.001	1.21	[0.827; 1.61]

*n (Phubbee) = 54, n (Phubber) = 62, n (Attentive Conversation) = 54.*

#### Mediation Analyses

In order to examine whether feelings of ostracism mediated the effect of condition on need satisfaction and mood, we conducted mediation analyses for the comparison between the phubbee condition and the attentive conversation condition as well as between the phubber condition and the attentive conversation condition with the R package mediation (Tingley et al., 2014).

#### Phubbee vs. Attentive Conversation

**FIGURE 1:**
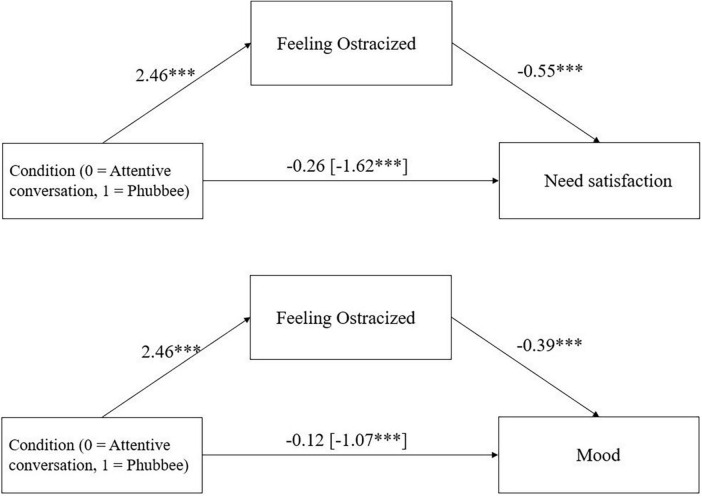
Mediation effect of condition (attentive conversation vs. phubbee) via feelings of ostracism on need satisfaction and mood in Study 1 including the direct effect of condition on need satisfaction and mood (with total effect in parentheses). *p < 0.05, **p < 0.01, ***p < 0.001.

Feelings of ostracism mediated the effect of condition on need satisfaction, *ab* = −1.35, 95%-CI [−1.71; 1.01], *p* < 0.001, and on mood, *ab* = −0.96, 95%-CI [−1.36; −0.57], *p* < 0.001 (Figure 1).

#### Phubber vs. Attentive Conversation

**FIGURE 2:**
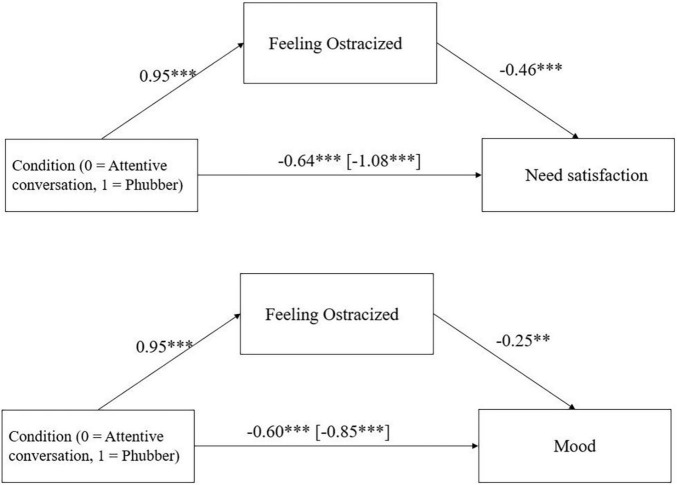
Mediation effect of condition (attentive conversation vs. phubber) via feelings of ostracism on need satisfaction and mood in Study 1 including the direct effect of condition on need satisfaction and mood (with total effect in parentheses). *p < 0.05, **p < 0.01, ***p < 0.001.

There was a partial mediation via feelings of ostracism of the effect of condition on need satisfaction, *ab* = −0.44, 95%-CI [−0.64; −0.28], *p* < 0.001 and on mood, *ab* = −0.24, 95% CI [−0.41; −0.09], *p* = 0.002 (Figure 2).

### Discussion

First of all, Study 1 replicated findings by Hales et al. (2018) and, thereby, provides further evidence for the assumption that phubbing can be perceived as ostracism. Participants who remembered a phubbing episode in which they were phubbed by another individual reported experiencing a threat to their fundamental needs, experienced negative mood and felt ostracized. Extending the findings by Hales et al. (2018), we also found that participants who remembered a situation in which they had phubbed someone else reported lower need satisfaction, more negative mood and greater feelings of ostracism compared to a control group. This is in line with research showing that ostracizers experience negative consequences of their behavior as well (Legate et al., 2013). However, these consequences might be less aversive for phubbers than for phubbees: Phubbers reported experiencing lower need threat and lower feelings of ostracism than did phubbees. Only regarding mood no significant difference between phubbees and phubbers emerged.

As argued by Giesen and Echterhoff (2018), this similarity in mood might be caused by the automatic operation of the ostracism detection system. Specifically, this system warns the individual when any sign of ostracism is detected by inducing negative mood. Compared to need threat, mood is an especially suitable warning signal because it has high informational value (Schwarz and Clore, 1983, 1996). Specifically, negative mood signals environmental threats or risks to fundamental goal achievement. Because phubbing can be perceived as an ostracism cue, it is no surprise that it activates the detection system, causing similar negative mood in phubbees and phubbers.

Even though the negative consequences for phubbers are plausible given prior findings on ostracism (Legate et al., 2013; Giesen and Echterhoff, 2018), they must be interpreted with caution. Specifically, given the retrospective nature of the present study, we do not know what motivated the phubbers to turn their attention to their mobile phones. While feelings of ostracism fully mediated the effect of the condition on both need satisfaction and mood for phubbees (vs. participants in the attentive conversation condition), they only partially mediated the effect of the condition on need satisfaction and mood for phubbers. It is likely that additional factors influenced phubbers mood and need satisfaction, and it is possible that negative mood and lower need satisfaction caused the phubbing behavior in the first place. Exploratory qualitative analyses of the texts written by our participants revealed that phubbers tended to use their mobile phones especially when they were bored or annoyed by the conversation, or when they had received a text message. Future research is needed to further investigate the circumstances under which individuals start interacting with their mobile phones, and when this might cause negative feelings for themselves.

Additionally, by asking participants to remember a past phubbing episode, the procedure of Study 1 may convey potential demand characteristics (Orne, 1962). Similarly, participants might have remembered their own intrapersonal consequences as being more negative than they actually were. To overcome these limitations, in Study 2 we manipulated phubbing in an ongoing conversation.

## Study 2

To examine effects of phubbing on need threat, mood, feelings of ostracism, and behavioral trust, we conducted another experiment in a standardized laboratory setting. Like Gonzales and Wu (2016), we manipulated phubbing (vs. neutral behavior) during a 10-min conversation in Study 2. However, in contrast to the study by Gonzales and Wu, phubbers in our study only focused their attention on the mobile phone for a short period of time and continued their conversation with the participant afterward. We thus ensured high external validity by manipulating phubbing more realistically (Misra et al., 2016). Additionally, we varied different relevant aspects of phubbing. More specifically, a confederate either interacted once vs. three times with her or his mobile phone during the conversation, initiated this interaction in a proactive vs. reactive way, and pretended to read a text message vs. to read and also answer it. Since we assume phubbing to be perceived as ostracism, we predicted that all kinds of phubbing affect need satisfaction, mood and feelings of ostracism. These variations of phubbing had not previously been varied systematically. We explored the effects of these variations in our analyses.

In addition to self-reported effects on fundamental needs, mood, and feelings of ostracism, we assessed, for the first time, behavioral consequences of phubbing. More precisely, we measured the phubbee’s behavioral trust toward the phubber by means of the trust game (Berg et al., 1995). Prior research on ostracism has demonstrated that ostracism causes a reduction of trust shown in this game (Hillebrandt et al., 2011). Therefore, we expect that phubbing will cause participants to display less behavioral trust when they were previously phubbed (vs. when they were not). Finally, to replicate and extend prior research on phubbing, we assessed different interpersonal variables that were previously shown to be negatively affected by phubbing (e.g., perceived politeness and attentiveness of the phubber; Vanden Abeele et al., 2016).

### Methods

#### Participants and Design

The design of Study 2 was a 2 (phubbing frequency: 1 vs. 3 times) × 2 (phubbing initiation: proactive vs. reactive) × 2 (modality: reading vs. writing) between-subjects design. Additionally, we recruited participants for a control condition (Attentive Conversation). Participants were randomly assigned to the conditions. To determine the required sample size, we calculated a power analysis with G*Power (Faul et al., 2009) based on the 2 × 2 × 2 design, with *d* = 0.71 (Hillebrandt et al., 2011), *1-*β = 0.95, and α = 0.05. The analysis revealed a required sample size of *N* = 106 participants. To be able to control for potential dropout or unusable data, we recruited an additional 5% of participants (i.e., *N* = 112). To make a meaningful comparison of main effects with the Attentive Conversation condition possible, an additional 52 participants (i.e., half of the *a priori* calculated samples size) were recruited for the Attentive Conversation condition. One participant was excluded from further analyses because of incomplete data due to computer problems. The final sample size consisted of *N* = 165 participants (118 female, 47 male), with a mean age of 23.88 years (*SD* = 5.37 years).

#### Procedure

Upon entering the laboratory, participants were introduced to the second alleged other participant (i.e., the confederate; the gender of the confederate was counterbalanced between participants). Both read and signed an informed consent. Afterward, they had a 10-min long conversation, which was videotaped. During this interaction, phubbing was manipulated. Thereafter, reflexive needs and mood were assessed and the trust game (Berg et al., 1995) was played via the computer. Furthermore, to replicate and extend previous findings on the consequences of phubbing, we assessed the following situationally influenced variables: politeness and attentiveness of the phubber (Vanden Abeele et al., 2016), self-reported trust, and inclusion of other in the self (Aron et al., 1997)^3^. Finally, potential moderators^4^ and demographic data were assessed. At the end of the study, participants were debriefed and received course credit or 4 € as compensation for their participation.

#### Materials

##### Phubbing Manipulation

Participants were told that the present study was conducted to investigate interpersonal processes in zero-acquaintance conversations and that they would have a conversation with another unacquainted participant for 10 min. In fact, this other participant was a male or female confederate who followed a prescribed script during the interaction. During the conversation, the participant and confederate were sitting in front of each other and their task was to answer 12 personal questions to induce self-disclosure (e.g., “What is your most treasured memory?”; Aron et al., 1997). The answers by the confederate were standardized and memorized. During the conversation, the confederate and participant were asked to take the time into account (i.e., 10 min) so that they could ideally discuss all questions. For that purpose, a clock was put on the table. When taking seat, the confederate always put his or her mobile phone down, with the display facing toward the table, to rule out effects of the mere presence of a mobile phone on perceived conversation quality (Przybylski and Weinstein, 2013). After 10 min of the conversation, the experimenter knocked on the door and allegedly led the confederate to a different room, where she or he could finish the rest of the experiment.

During the conversation, phubbing was manipulated. To manipulate the frequency of phubbing, the confederate either used his or her mobile phone once (1x Phubbing condition) after about 6 min or three times (3x Phubbing condition; after about 3, 6, and 9 min). To manipulate the initiation of phubbing, the experimenter either sent a message at the predefined times to the confederate, causing the ringtone of their mobile phone to sound (reactive initiation) or did not send a message (proactive condition). In the reactive phubbing condition, the confederate picked up the phone only after the ringtone. In the proactive phubbing condition, no ringtone sounded, so the confederate self-initiated the phone interaction. The modality of phubbing was then manipulated by pretending to read a message or by reading as well as typing a message. The average duration of phubbing was 10.70 s (*SD* = 5.51) in the 1x Phubbing condition and 11.93 s (*SD* = 8.41) per phubbing in the 3x Phubbing condition. In the Attentive Conversation condition, the confederate drank three times from his or her water bottle (after about 3, 6, and 9min). The average duration of drinking water was 3.46 s (*SD* = 1.97). The total duration of 3x drinking did not differ significantly from the duration of 1x Phubbing, *t*(76.20) = 0.25, *p* = 0.800, *d* = 0.06. It did differ from the total duration of 3x Phubbing, *t*(57.21) = 6.94, *p* < 0.001, *d* = 1.23.

##### Need Satisfaction, Mood and Feelings of Ostracism

Need satisfaction was assessed by means of the same adapted need-threat scale already used in Study 1 (α = 0.87; Williams, 2009). Mood (α = 0.79) and feelings of ostracism (*r*_*SB*_ = 0.88; Williams, 2009) were also assessed as in Study 1, except that the PANAS (Krohne et al., 1996) was not included in the present study. Thus, mood was only assessed by means of the eight items by Williams (2009) for the sake of parsimony.

##### Trust Game

Behavioral trust was measured by means of an adapted version of the trust game (Berg et al., 1995). In this game, the participant was informed that they and the confederate would be randomly given the role of a sender or receiver of lots for vouchers of an online shop. In reality, the participant was always the sender. They received ten lots and had to decide how many lots she or he wanted to send to the confederate. Before this decision, they were informed that the chosen amount would be tripled and given to the confederate, who in turn would decide how many lots they want to send back to the participant. This included the option to send back no lots. Thus, it was possible that the participant (the sender) could end up with fewer lots than before if the recipient sent back no lots or too few lots. On the contrary, the participant could increase her or his number of lots if he or she trusted the confederate to send back enough lots. Therefore, the amount of sent lots is an index of the level of trust the participant has in the confederate.

##### Other Situational Variables

Perceived politeness and attentiveness of the phubber was assessed as by Vanden Abeele et al. (2016). Specifically, perceived politeness was assessed by three items (e.g., “My conversation partner behaved inappropriately”, α = 0.84). Attentiveness of the phubber was measured by four items (e.g., “My conversation partner seemed involved with the conversation”, α = 0.88). For both scales, participants were asked to indicate on 7-point Likert scales to what extent they agreed with each statement (1 = *I don’t agree*; 7 = *I totally agree*).

### Results

#### Need Satisfaction

To investigate the effect of the different phubbing types on need satisfaction, we conducted a 2 (frequency: 1 vs. 3 times) × 2 (initiation: proactive vs. reactive) × 2 (modality: reading vs. writing) ANOVA. The ANOVA revealed a marginally significant effect of Frequency on need satisfaction *F*(1, 103) = 3.34, *p* = 0.071, η*_*p*_*^2^ = 0.03, 90% CI = [0;0.1]. Participants who were phubbed three times (*M* = 3.80, *SD* = 0.50) tended to report less need satisfaction than those who were phubbed only once (*M* = 3.96, *SD* = 0.40). No interaction or other main effects were (marginally) significant (all *F*s < 1.30, all *p*s> 0.265; see Table 4 for the descriptive statistics).

**TABLE 4:** Mean need satisfaction (SDs in parentheses) as a function of modality of phubbing (reading vs. writing), initiation of phubbing (proactive vs. reactive), and phubbing frequency.

		Need satisfaction	
		
		Frequency	
		
Modality	Initiation	1x	3x
Reading	Proactive	4.04 (0.24)	3.79 (0.55)
	Reactive	4.05 (0.47)	3.82 (0.57)
Writing	Proactive	3.96 (0.42)	3.89 (0.47)
	Reactive	3.79 (0.42)	3.82 (0.57)

*n (1x/Reading/Proactive)=14, n (1x/Reading/Reactive)=14, n (3x/Reading/Proactive)=15, n (3x/Reading/Reactive)=13, n (1x/Writing/Proactive)=14, n (1x/Writing/Reactive)=13, n (3x/Writing/Proactive)=13, n (3x/Writing/Reactive)=15. Descriptive statistics of the Attentive Conversation condition: M=3.90, SD=0.46.*

#### Mood

Another ANOVA with our independent variables was conducted to investigate the effects on mood. However, the analysis revealed no significant effects (all *F*s < 2.70, *p*s > 0.105; see Supplementary Material for the descriptive statistics).

#### Feelings of Ostracism

Here, an ANOVA revealed that frequency of phubbing significantly affected feelings of being ignored and excluded, *F*(1, 103) = 16.51, *p* < 0.001, η*_*p*_*^2^ = 0.148, 90% CI = [0.05;0.23]. Participants in the 3x Phubbing condition felt more ignored and excluded (*M* = 1.88, *SD* = 1.11) than those in the 1x Phubbing condition (*M* = 1.20, *SD* = 0.47; all other *F*s < 1.00, all other *p*s > 0.320, see Table 5 for the descriptive statistics).

**TABLE 5:** Mean feelings of ostracism (SDs in parentheses) as a function of modality of phubbing (reading vs. writing), initiation of phubbing (proactive vs. reactive), and phubbing frequency.

		Feelings of ostracism	
		
		Frequency	
		
Modality	Initiation	1x	3x
Reading	Proactive	1.07 (0.27)	1.87 (1.29)
	Reactive	1.23 (0.56)	1.77 (1.22)
Writing	Proactive	1.32 (0.58)	1.96 (0.95)
	Reactive	1.15 (0.38)	1.96 (1.06)

*n (1x/Reading/Proactive)=14, n (1x/Reading/Reactive)=14, n (3x/Reading/Proactive)=15, n (3x/Reading/Reactive)=13, n (1x/Writing/Proactive)=14, n (1x/Writing/Reactive)=13, n (3x/Writing/Proactive)=13, n (3x/Writing/Reactive)=15. Descriptive statistics of the Attentive Conversation condition: M=1.02, SD=0.14.*

#### Trust Game

To investigate the effects on the trust game, we calculated another ANOVA. The analysis revealed a significant effect of Frequency on the amount of sent lots, *F*(1, 103) = 4.88, *p* = 0.029, η*_*p*_*^2^ = 0.05, 90% CI = [0.002;0.12]. Participants sent fewer lots to their conversation partner when they were phubbed three times (*M* = 7.09, *SD* = 2.79) compared to only once (*M* = 8.16, *SD* = 2.59). In addition, there was a marginally significant effect of the initiation of phubbing, *F*(1, 103) = 3.61, *p* = 0.060, η*_*p*_*^2^ = 0.03, 90% CI = [0;0.10]. Participants tended to send fewer lots to their conversation partner when he or she phubbed reactively (*M* = 7.11, *SD* = 2.62) vs. proactively (*M* = 8.13, *SD* = 2.78). No other significant effects emerged (all *F*s < 2.70, *p*s > 0.100; see Table 6 for the descriptive statistics). In sum, these findings indicate that the frequency of phubbing decreases participants’ trust in the phubber. Furthermore, reactive phubbing seems to be slightly more negative than proactive phubbing.

**TABLE 6:** Mean lots sent to partner (SDs in parentheses) as a function of modality of phubbing (reading vs. writing), initiation of phubbing (proactive vs. reactive), and frequency of phubbing.

		Lots	
		
		Frequency	
		
Modality	Initiation	1x	3x
Reading	Proactive	8.36 (2.50)	8.73 (2.66)
	Reactive	7.43 (2.56)	6.46 (3.07)
Writing	Proactive	8.64 (2.95)	6.62 (2.79)
	Reactive	7.81 (2.50)	6.43 (2.59)

*n (1x/Reading/Proactive)=14, n (1x/Reading/Reactive)=14, n (3x/Reading/Proactive)=15, n (3x/Reading/Reactive)=13, n (1x/Writing/Proactive)=14, n (1x/Writing/Reactive)=13, n (3x/Writing/Proactive)=13, n (3x/Writing/Reactive)=15. Descriptive statistics of the Attentive Conversation condition: M=8.00, SD=2.94.*

#### Politeness

For politeness, there was a significant effect of Frequency, *F*(1, 110) = 12.17, *p* = 0.001, η*_*p*_*^2^ = 0.11, 90% CI = [0.03;0.19]. Conversation partners who phubbed three times were perceived as less polite (*M* = 5.45, *SD* = 1.66) than those who phubbed once (*M* = 6.37, *SD* = 0.97). There were no other significant interactions or main effects on politeness (all *F*s < 1.40, all *p*s > 0.240).

#### Attentiveness

For attentiveness, the ANOVA also revealed a significant effect of Frequency, *F*(1, 103) = 11.82, *p* = 0.001, η*_*p*_*^2^ = 0.10, 90% CI = [0.03; 0.19]. Participants who were phubbed three times (*M* = 5.56, *SD* = 1.24) vs. once (*M* = 6.25, *SD* = 0.77) rated their partner to be less attentive. All other effects were not significant (all *F*s < 1, all *p*s > 0.320).

#### Comparison With the Attentive Conversation Condition

To further investigate the consistently found effect of the frequency of phubbing, we conducted one-way ANOVAs for each dependent variable comparing the Attentive Conversation condition, the 3x Phubbing condition, and the 1x Phubbing condition. For *post hoc* tests, we applied the Bonferroni correction so that we interpret *p* < 0.016 as significant.

##### Feelings of Ostracism

There was a significant effect of the condition on feelings of ostracism, *F*(2, 160) = 22.15, *p* < 0.001, η*_*p*_*^2^ = 0.22. *Post hoc* t-tests revealed that participants in the 3x Phubbing condition felt more ignored and excluded (*M* = 1.88, *SD* = 1.11) than those in the 1x Phubbing condition (*M* = 1.20, *SD* = 0.47) who felt more excluded than participants in the Attentive Conversation condition (*M* = 1.02, *SD* = 0.14), all | *t*| s > 2.75, all *p*s < 0.008.

##### Politeness

There was a significant main effect of the condition on perceptions of politeness, *F*(2, 160) = 16.97, *p* < 0.001, η*_*p*_*^2^ = 0.18. Participants experienced their partner to be less polite in the 3x Phubbing condition (*M* = 4.78, *SD* = 1.66) than in the 1x Phubbing condition (*M* = 5.70, *SD* = 0.97), *t*(88.74) = −3.59, *p* < 0.001, and in the Attentive Conversation condition (*M* = 6.025, *SD* = 0.480), *t*(64.61) = 5.385, *p* < 0.001. The difference in the perceived politeness between the Attentive Conversation condition and the 1x Phubbing condition was not significant under the Bonferroni-adjusted alpha level, *t*(79.76) = 2.207, *p* = 0.030.

##### Attentiveness

There was a significant main effect of condition on ratings of attentiveness, *F*(2, 160) = 9.53, *p* < 0.001, η*_*p*_*^2^ = 0.106. Participants in the 3x Phubbing condition rated their partner as less attentive (*M* = 5.56, *SD* = 1.24) than participants in the 1x Phubbing condition (*M* = 6.25, *SD* = 0.77), *t*(91.83) = −3.54, *p* < 0.001, and in Attentive Conversation condition (*M* = 6.24, *SD* = 0.77), *t*(89.27) = 3.502, *p* < 0.001. There was no significant difference between the Attentive Conversation condition and the 1x Phubbing condition, *t*(105.95) = 0.01, *p* = 0.922.

##### Need Satisfaction, Mood, and Trust Game

There was no significant main effect of the condition on need satisfaction, mood, or the trust game (all *F*s < 2.41, all *p*s > 0.093).

#### Mediation Analyses

In order to examine whether there were indirect effects of the condition on need satisfaction, mood, and trust in the trust game via feelings of ostracism, we conducted mediation analyses with the R package mediation (Tingley et al., 2014) for 1x phubbing vs. 3x phubbing, 1x phubbing vs. attentive conversation, and 3x phubbing vs. attentive conversation.

##### 1x Phubbing vs. 3x Phubbing

**FIGURE 3:**
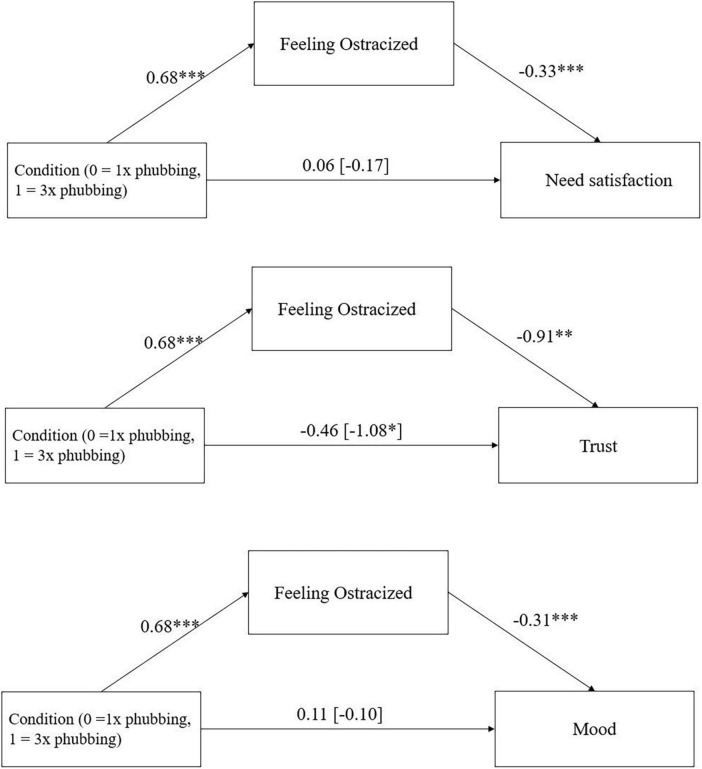
Mediation effect of condition (1x phubbing vs. 3x phubbing) via feelings of ostracism on need satisfaction, trust in the trust game, and mood in Study 2 including the direct effect of condition on need satisfaction and mood (with total effect in parentheses). *p < 0.05, **p < 0.01, ***p < 0.001.

There was a significant indirect effect of condition via feelings of ostracism on need satisfaction, *ab* = −0.23, 95%-CI [−0.35; −0.11], *p* < 0.001,. amount of lots sent in the trust game, *ab* = −0.62, 95%-CI [−1.17; −0.19], *p* = 0.002, and mood, ab = −0.21, 95%-CI [−0.33; −0.10], *p* < 0.001 (Figure 3).

##### Attentive Conversation vs. 1x Phubbing

**FIGURE 4:**
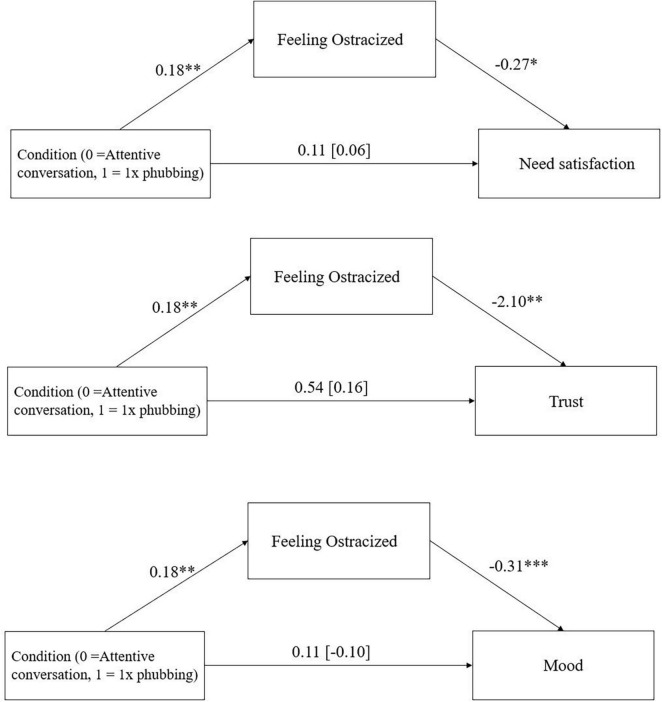
Mediation effect of condition (attentive conversation vs. 1x phubbing) via feelings of ostracism on need satisfaction, trust in the trust game, and mood in Study 2 including the direct effect of condition on need satisfaction and mood (with total effect in parentheses). *p < 0.05, **p < 0.01, ***p < 0.001.

There was a significant indirect effect of condition via feelings of ostracism on need satisfaction, *ab* = −0.05, 95%-CI [−0.12; −0.003], *p* = 0.031,. amount of lots sent in the trust game, *ab* = −0.38, 95%-CI [−0.83; −0.06], *p* = 0.013, and mood, *ab* = −0.06, 95%-CI [−0.13; −0.01], *p* = 0.013 (Figure 4).

##### Attentive Conversation vs. 3x Phubbing

**FIGURE 5:**
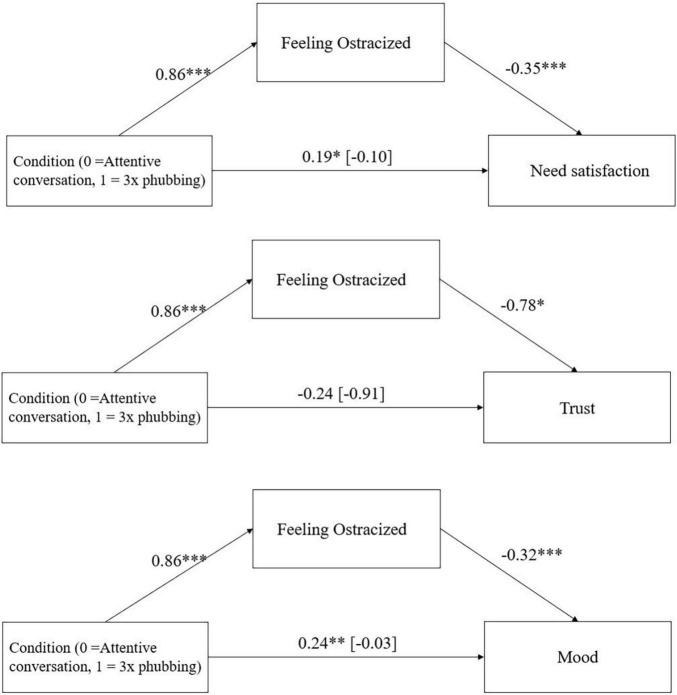
Mediation effect of condition (attentive conversation vs. 3x phubbing) via feelings of ostracism on need satisfaction, trust in the trust game, and mood in Study 2 including the direct effect of condition on need satisfaction and mood (with total effect in parentheses). *p < 0.05, **p < 0.01, ***p < 0.001.

There was a significant indirect effect of condition via feelings of ostracism on need satisfaction, *ab* = −0.30, 95%-CI [−0.44; −0.18], *p* < 0.001, amount of lots sent in the trust game, *ab* = −0.67, 95%-CI [−1.34; −0.10], *p* = 0.022, and mood, *ab* = −0.27, 95%-CI [−0.40; −0.16], *p* < 0.001 (Figure 5).

### Discussion

Study 2 revealed that the frequency of phubbing influences the extent of its negative consequences. Specifically, when participants were phubbed three times (vs. once) during the conversation, participants experienced slightly less need satisfaction and felt more ostracized by their conversation partner. However, there was no significant difference between both the 3x and 1x phubbing condition and the Attentive Conversation condition on the fundamental needs. Still, participants who experienced phubbing reported to feel more ignored and excluded than those who engaged in an attentive conversation, replicating Gonzales and Wu’s (2016) as well as McDaniel and Wesselmann’s (2021) findings. Furthermore, participants who were phubbed three times felt even more ignored and excluded than participants who were phubbed only once.

Possibly, the missing difference in the conditions regarding fundamental needs can be explained by the operation of an automatic ostracism-detection system (Spoor and Williams, 2007; Kerr and Levine, 2008). This system detects all minor cues of ostracism in the environment and alarms the individual by inducing social pain. Our confederates in the Attentive Conversation condition were instructed to drink water three times, and when doing so, they were likely directing their eye gaze away from the participants. Since prior research has shown that averted eye gaze can be perceived as a minor form of ostracism and is sufficient to induce need threat (Wirth et al., 2010), this might explain the lack of differences in the present study. Therefore, we nevertheless conclude that Study 2 provides further evidence for the assumption that phubbing can be perceived as ostracism, with similar consequences for the individuals’ well-being.

Furthermore, as already shown in previous studies (Vanden Abeele et al., 2016), phubbing has negative effects on interpersonal variables. When the phubbee was phubbed three times (vs. once), he or she regarded the phubber as less attentive and polite. Attentiveness and politeness were thwarted more by phubbing than by drinking water. These negative effects of phubbing were also found in the trust game: When participants were phubbed three times (vs. once), they sent fewer lots to the phubber.

Next to the effects of the frequency of phubbing, the initiation type of phubbing (proactive vs. reactive) also tended to affect behavioral trust shown toward the phubber. More precisely, reactive (vs. proactive) phubbing tended to reduce the trustworthiness of the phubber in the trust game. However, these findings are not consistent with prior research (Vanden Abeele et al., 2016). Therefore, future research is needed to further investigate the consequences of reactive vs. proactive phubbing and its underlying mechanisms. Even though Vanden Abeele and colleagues have argued that reactive phubbing is more socially accepted, reactive phubbing might be more strongly perceived as an aversive or impolite interruption of the conversational flow given the sound of the ringtone. Additionally, reactive phubbing clearly indicates that the phubber reacts to another person and thus excludes the phubbee from a virtual conversation. When the phubbing is proactive, it is unclear whether the phubber is reacting to another person or is doing something else on her or his mobile phone.

We found significant indirect effects via feelings of ostracism on need satisfaction, lots sent in the trust game, and mood for all comparisons, although only few analyses showed significant total effects of the conditions. The significant indirect effects fit with our reasoning that phubbing increases feelings of ostracism which in turn reduce need satisfaction, positive mood, and trust in the phubber. The failure to detect total effects of the conditions on the dependent variables was possible due to power issues. Therefore, future research should aim to replicate these findings with larger samples.

## General Discussion

The present research replicated and extended prior research on the consequences of phubbing on mood, need threat, and feelings of ostracism (Gonzales and Wu, 2016; Chotpitayasunondh and Douglas, 2018; Hales et al., 2018; McDaniel and Wesselmann, 2021). We found that phubbing induces feelings of being ostracized (Study 1 and 2), which threatens fundamental needs, causes negative mood (Study 1 and 2), and reduces behavioral trust (Study 2). Importantly, this does not merely hold for phubbees, but also for phubbers (Study 1). However, the frequency of phubbing appears to play an important role. The more often individuals were phubbed in Study 2, the more need threat and feelings of ostracism they experienced. The frequency of phubbing further affected the phubbee’s behavioral trust toward the phubber. When phubbed three times (vs. once), phubbees tended to send fewer lots to their phubbers. However, there was only a difference between the Phubbing conditions and the Attentive Conversation condition (where the confederate drank water three times) for feelings of ostracism, politeness, and attentiveness. Potential reasons for missing differences are discussed below.

Prior research on whether the usage of mobile phones affects interpersonal relationships has mainly been correlational, revealing an association between phubbing and conversational quality as well as relationship quality (Cameron and Webster, 2011; Klein, 2014; Misra et al., 2016; Roberts and David, 2016; Bröning and Wartberg, 2022). Some experiments have focused on impression formation and conversational quality, showing that the presence and usage of mobile phones reduces conversational quality and has a negative effect on the phubbee’s impression of the phubber (Przybylski and Weinstein, 2013; Vanden Abeele et al., 2016). Gonzales and Wu (2016) and Hales et al. (2018) as well as Chotpitayasunondh and Douglas (2018) were the first to demonstrate that phubbing can be perceived as ostracism. Our research replicates the findings of Hales et al. (2018) as well as Chotpitayasunondh and Douglas (2018) by showing that remembering a past phubbing episode or imagining being phubbed causes feelings of being ostracized, negative mood and need threat. Moreover, Gonzales and Wu (2016) showed that an experimentally induced phubbing episode during an ongoing conversation induces feelings of being ostracized. Our research extends these findings by showing that phubbing also has negative consequences for phubbers. In addition, by studying behavior in the trust game, we provided the first evidence on how individuals behave toward those who use their mobile phone in the presence of others.

Phubbers divide their attention between their mobile phone and the physically present interaction partner and likely exclude her or him from a digital interaction (Klein, 2014; Vanden Abeele et al., 2016). The innate ostracism detection system alarms the individual automatically when any minor cue of ostracism, including phubbing, is detected by causing immediate social pain (Kerr and Levine, 2008).

Given this social pain inflicted by phubbing, it is not surprising that phubbing also negatively affects behavior shown toward the phubber. Research on ostracism has already demonstrated that ostracized individuals show less behavioral trust toward their ostracizers than included individuals and send them fewer lots in the trust game (Hillebrandt et al., 2011). Our second study replicates this finding and extends it to phubbing. Thus, phubbees exhibit lower trust in phubbers when they are phubbed more frequently.

Overall, our present research demonstrates that we can learn about phubbing by deriving knowledge from the existing ostracism literature. But also research on inattentive listening can additionally help us to understand the consequences of phubbing. For example, it has been shown that narrators reduce the quantity and quality of what they tell their listener when they are interacting with an inattentive listener (see Pasupathi and Rich, 2005), narrators have worse memory for what they were talking about (Pasupathi et al., 1998; Pasupathi and Hoyt, 2010), and reduce their self-verification during the conversation (Pasupathi and Rich, 2005). Correlational research has further shown that perceived listening quality is related to perceived sympathy of the conversation partner, trust in her or him, and the mood of the narrator (Lloyd et al., 2015). Thus, when phubbing is perceived as inattentive listening, this might also partially explain our findings. This could also explain why there were fewer differences between the Phubbing conditions and the Attentive Conversation condition because the confederate in the Attentive Conversation condition might also have appeared inattentive when she or he drank from her or his water bottle. Yet, participants felt more ostracized in the 1x Phubbing condition than in the Attentive Conversation condition, even though the total duration of breaks in the conversation did not differ between these two conditions. Thus, the finding that participants felt more excluded in the phubbing conditions than in the Attentive Conversation condition cannot merely be explained by the fact that confederates were distracted from the conversation for a longer time period in the phubbing than in the Attentive Conversation condition.

### Limitations and Future Research

One limitation of the present line of research is the retrospective nature of Study 1. Since our participants were asked to remember a past phubbing episode, it is unclear whether phubbing really has negative consequences for the phubbers’ well-being and causes their need threat and negative mood. Alternatively, this need threat and negative mood might have been the reason why they initiated the phubbing in the first place. Various reasons for phubbing behavior have been discussed, ranging from social media addiction to social anxiety (Rahman et al., 2022) as well as negative emotions such as boredom or fear of missing out (Al−Saggaf and O’Donnell, 2019). With our correlational data, we cannot determine whether negative mood was an antecedent or consequence of phubbing for phubbers. However, research demonstrating negative effects of ostracism for ostracizers provide support for the conclusion that phubbing may have caused phubbers’ negative mood and need threat (Legate et al., 2013). In addition, our confederates in Study 2 repeatedly complained about the aversive experience of phubbing someone, similar to confederates in a study by Williams and Sommer (1997) who were instructed to ostracize participants in a ball tossing game. Nevertheless, future research is needed to provide further evidence for the aversive consequences of phubbing for the phubber. For example, in future research participants’ well-being could be assessed after they were instructed to phub another person in the lab (see Vanden Abeele et al., 2016).

Another limitation is that we could not standardize the depth of the conversation in which phubbing occurred. Of course, the conversation topics in Study 2 were standardized, yet the depth of the answers given by the participants might have varied. Probably, phubbing is perceived as even more inappropriate when the conversation becomes less shallow, more personal, and more elaborate (Przybylski and Weinstein, 2013). We suspect that phubbing has even more negative effects on personal well-being, trust and the willingness to cooperate for less superficial, more personally engaging conversations. Future research could compare the effects of phubbing between such levels of conversations.

Also, phubbing occurs more often between people who knew each other before such as friends, partners, and family members than between strangers (Al-Saggaf and MacCulloch, 2019). The observed effects of phubbing could vary depending on relationship closeness with the interaction partner. Future research could compare effects of phubbing by a stranger with phubbing by a closer interaction partner. Another limitation of Study 2 is that we did not examine whether the gender composition of the pairs had an impact on the effects of phubbing due to power limitations. Previous research shows that participants are more competitive in bargaining games when played with participants of the same gender (Sutter et al., 2009). Thus, gender composition could also have impacted participants’ behavior in the trust game. While we controlled for possible gender effects by counterbalancing the gender of the confederate between conditions, future research could examine whether there are interaction effects of the phubbing condition and gender composition regarding behavior in the trust game.

There are also limitations concerning our Attentive Conversation condition in Study 2 (i.e., 3x drinking water). First, this condition might have been too conservative to serve as a suitable control. Specifically, when drinking water, our confederates likely averted their eye-gaze away from our participants. Prior research on averted eye contact has demonstrated that this is sufficient to induce feelings of ostracism and need threat (Wirth et al., 2010). Thus, since reduced eye contact induces social pain, it is reasonable that there were no significant differences between our Phubbing conditions and the Attentive Conversation condition for need satisfaction and behavioral trust. Future research should implement a control group in which no or fewer cues of ostracism are present.

Second, one might argue that the duration of drinking in the Attentive Conversation condition was too short in comparison with the total duration of phubbing in the 3x Phubbing condition. Thus, significant differences between these two conditions on feelings of ostracism, perceived politeness and alertness could be explained by the duration of the conversational interruption. However, the total duration of drinking did not differ from the duration of phubbing in the 1x Phubbing condition. If the duration of the conversational interruption would have been an underlying mechanism of our effects, the 1x Phubbing condition and the 3x water drinking in the Attentive Conversation condition should both be significantly different from the 3x Phubbing condition on our dependent variables. In addition, we found a difference between the 1x Phubbing condition and the Attentive Conversation condition on feelings of ostracism and politeness. Thus, there must be another mechanism explaining the lack of significant differences with the control group on our main dependent variables. Future research is needed to identify this mechanism.

Finally, there are limitations regarding the power of Study 2. In calculating the required sample size, we assumed a large effect of the phubbing conditions. However, the design was quite complex for our sample size and the study was therefore underpowered to conduct potential smaller effects of the quite subtle variations in phubbing behavior. It is thus also possible that the failure to detect differences between conditions regarding need satisfaction was due to the study being underpowered. Although this type of study with confederates in the laboratory is quite labor intensive, future research should nevertheless aspire to further examine effects of phubbing behavior in larger samples.

## Conclusion

The present research demonstrated that phubbing has similar negative consequences as ostracism by threatening fundamental human needs and inducing negative mood. However, these negative consequences backfire on the phubber: Individuals who were phubbed more often, showed less behavioral trust toward their phubbers, which reduces the phubbers’ chances of gaining the benefits they can usually draw from interpersonal interactions. It is important that we are aware of the negative consequences of phubbing—an omnipresent and seemingly subtle behavior—which is increasingly gaining normative acceptance (Chotpitayasunondh and Douglas, 2016). Only by knowing the consequences can we deliberately choose how we want to treat and affect our conversation partners and influence the impression we make. One possible intervention for preventing phubbing would be to remind potential phubbers of the negative, backfiring effects of phubbing. Chronically accessible memories for such negative reverberations would deter potential phubbers from repeating acts of phubbing. Our research shows that it is worth considering the behavioral option put forward by an Australian campaign: “Stop phubbing” (Stop Phubbing, n.d.).

## Data Availability Statement

The raw data supporting the conclusions of this article will be made available by the authors, without undue reservation.

## Ethics Statement

Ethical review and approval was not required for the study on human participants in accordance with the local legislation and institutional requirements. The participants provided their written informed consent to participate in this study.

## Author Contributions

JK, AG-L, and GE designed the studies and wrote the manuscript. AG-L performed the studies. AG-L and JK analyzed the data. All authors contributed to the article and approved the submitted version.

## Conflict of Interest

The authors declare that the research was conducted in the absence of any commercial or financial relationships that could be construed as a potential conflict of interest.

## Publisher’s Note

All claims expressed in this article are solely those of the authors and do not necessarily represent those of their affiliated organizations, or those of the publisher, the editors and the reviewers. Any product that may be evaluated in this article, or claim that may be made by its manufacturer, is not guaranteed or endorsed by the publisher.

## Footnotes

1 Before assessing the moderator variables, we asked open-ended questions on potential reasons for the phubbing, the content of the conversation, the behavioral response of the participant and the conversation partner. However, these questions are not considered in the present manuscript and are therefore not explained in detail.

2 In total, we measured ten potential moderators: emotional closeness to the conversation partner, deepness of the conversation, degree of self-disclosure, duration of phubbing relative to the total duration of the conversation, frequency of phubbing, proactive vs. reactive phone use, announcement of the phone use, excusing the phone use, attachment anxiety (Thomson et al., 2012) and the need to belong (Hartung and Renner, 2014). To control for an inflation of the Type I error rate, the critical alpha level was adjusted by means of the Bonferroni correction (Holm, 1979; α = 0.05/10 = 0.005). Considering this adjusted critical alpha level, none of the moderation analyses were significant (all *F*s < 2.70; *p*s > 0.010). Therefore, we refrain from explaining these moderators in detail. In addition to the above-mentioned moderators, we further assessed as potential moderators the agreeableness (Rammstedt et al., 2013) and social norms of phubbing (Chotpitayasunondh and Douglas, 2016). However, given their low internal reliability (αs < 0.38), no further analyses were calculated with these variables.

3 See Supplementary Material for a description of the measures and the respective findings.

4 In total, we measured eight moderators in the second study: agreeableness (Rammstedt et al., 2013), attachment anxiety (Thomson et al., 2012), self-esteem (Von Collani and Herzberg, 2003), need to belong (Hartung and Renner, 2014), experience with being phubbed by others, experience with phubbing others, and injunctive and descriptive social norms of phubbing (Chotpitayasunondh and Douglas, 2016). Since the internal reliability of the descriptive and injunctive norm questionnaires (α = 0.39 and α = 0.21) as well as the agreeableness scale (α = 0.46) were too low, these scales were not considered in further analyses. Furthermore, to prevent an inflation of the Type I error rate for the remaining moderation analyses, the critical alpha level was adjusted by means of the Bonferroni correction (Holm, 1979; α = 0.05/5 = 0.01). The analyses revealed no significant moderations except for experience with phubbing others (all other *F*s < 5.60; all other *p*s ≥ 0.020). This variable is described in detail in Supplementary Material.
