# Corrigendum: Feeling ostracized by others' smartphone use: the effect of phubbing on fundamental needs, mood, and trust

**DOI:** 10.3389/fpsyg.2023.1213753

**Published:** 2023-06-13

**Authors:** Judith Knausenberger, Anna Giesen-Leuchter, Gerald Echterhoff

**Affiliations:** Department of Psychology, University of Münster, Münster, Germany

**Keywords:** phubbing, ostracism, fundamental needs, mood, interpersonal relations, trust game

In the published article, there was an error in the Funding statement, which originally stated “We acknowledge support from the Open Access Publication Fund of the University of Münster.”

The correct Funding statement appears below.

