# Supplementary material for: Feeling Ostracized by Others’ Smartphone Use: The Effect of Phubbing on Fundamental Needs, Mood, and Trust
Source: Front Psychol. 2022 Jul 1;13:883901. doi: 10.3389/fpsyg.2022.883901 (PMC9285876; doi:10.3389/fpsyg.2022.883901)
Supplement: Supplementary file 1 [file Presentation_1.pdf]

Supplemental Material

Study 2

Descriptive statistics of the effect of the Modality of Phubbing, the Initiation of Phubbing and Frequency of Phubbing on Mood.

Table S1: Mean Mood (*SD*s in parentheses) as a Function of Modality of Phubbing (Reading vs. Writing), Initiation of Phubbing (Proactive vs. Reactive) and Frequency of Phubbing.

		Mood	
		Frequency	
Modality	Initiation	1x	3x
Reading	Proactive	4.545 (0.297)	4.358 (0.511)
	Reactive	4.475 (0.426)	4.269 (0.453)
Writing	Proactive	4.411 (0.513)	4.375 (0.497)
	Reactive	4.154 (0.347)	4.232 (0.497)

Note: *n* (1x/Reading/Proactive) = 14, *n* (1x/Reading/Reactive) = 15, *n* (3x/Reading/Proactive) = 15, *n* (3x/Reading/Reactive) = 13, *n* (1x/Writing/Proactive) = 14, *n* (1x/Writing/Reactive) = 13, *n* (3x/Writing/Proactive) = 13, *n* (3x/Writing/Reactive) = 14. Descriptive statistics of the Attentive Conversation condition: $M = 4.339$, $SD = 0.392$.

Other dependent variables assessed in Study 2

Self-reported trust. Self-reported trust was assessed by two items of the relatedness subscale of the Intrinsic Motivation Inventory (e.g., “I really feel like I could trust this person”, α = .79; Kooiman et al., 2015; McAuley et al., 1989). Answers were indicated on 5-point Likert scales (1: I don’t agree; 5: I totally agree). The ANOVA with our independent variables and self-reported trust as dependent variable revealed a marginally significant effect of Frequency, $F(1, 103) = 3.125$, $p = .080$, $\eta_p^2 = .029$, 90% CI = [0; .095]. Participants in the 3x Phubbing condition tended to report less trust in the phubber ($M = 3.268$, $SD = 0.986$) than those in the 1x Phubbing condition ($M = 3.582$, $SD = 0.768$). However, there was no significant difference between 3x Phubbing and drinking water ($M = 3.442$, $SD = 0.884$),

$t(105.87)$= -0.969, p = .334, nor between 1x Phubbing and drinking water, $t(101.15)$ = 0.869, p = .387.

Inclusion of other in the self. Inclusion of other in the self was assessed by means of Aron and colleagues' (1992) scale. Participants were asked to select one of seven increasingly overlapping circle pairs to represent the closeness between them and the confederate. A 2x2x2 ANOVA with our independent variables and the inclusion of other in the self as dependent variable revealed a significant interaction of the frequency and initiation of phubbing on inclusion of other in the self, $F(1, 103) = 4.224$, $p = .042$, $\eta_p^2 = .039$, 90% CI = [.001; .110]. Pairwise comparisons revealed that phubbers who phubbed three times were included more strongly in the self when they interacted proactively with their smart phone (M = 3.180, SD = 1.278) compared to reactively (M = 2.43, SD = 1.034; ΔM = 0.787, SE = .303, p = .011, d = -0.645, 95% CI = [-1.182; - 0.108]). Additionally, the interaction between Frequency and Modality reached significance, $F(1, 103) = 4.625$, $p = .034$, $\eta_p^2 = .043$, 90% CI = [.002; .116]. Pairwise comparisons showed that phubbers who phubbed three times tended to be more strongly included in the self when they typed (M = 3.040, SD = 1.261) vs. read a message (M = 2.570, SD = 1.136; ΔM = 0.521, SE = .303, p = .089, d = -0.392, 95% CI = [-0.920; 0.137]). The ANOVA revealed no other significant effects (all Fs < 2, ps > .110). Thus, more active and self-initiated phubbing led to stronger inclusion of other in the self.

Moderating variables assessed in Study 2

Experience with phubbing. To assess the participants' phubbing experience in their daily lives, they were asked how often they phub others in one day, and how often they are phubbed by others in one day. Both types of phubbing experiences were assessed by means of Likert scale items ranging from 1 (never) to 5 (often). Both items were considered separately as moderators, since Cronbach's alpha was too low for combining them into a single index of phubbing experience (α = .46). The variable "amount of phubbing others" moderated the effect of Initiation on the fundamental needs, $F(1, 95) = 7.979$, $p = .006$, $\eta_p^2 = .077$, 90% CI =

[.012; .154] (see Figure S1). For participants who reported a high amount of phubbing others (*M* + 1 *SD*) the analysis revealed a significant effect of Initiation type, $F(1, 95) = 7.676$, $p = .007$, $\eta_p^2 = .075$, 90% CI = [.011; .154]. When phubbing took place reactively, participants reported less need satisfaction compared to proactive phubbing (estimated marginal means: $M_{reactive\ =}$ 3.974, $SE_{reactive}$ = .090; $M_{proactive\ =}$ 4.008, $SE_{proactive}$ = .091). For participants who reported a low amount of phubbing others (*M* – 1 *SD*) the effect of Initiation type was not significant, $F(1, 95) = 1.827$, $p = .180$, $\eta_p^2 = .019$, 90% CI = [0; .075].

Figure S1. The effect of the Initiation of Phubbing on Need Satisfaction Depending on the Amount of Phubbing Others.

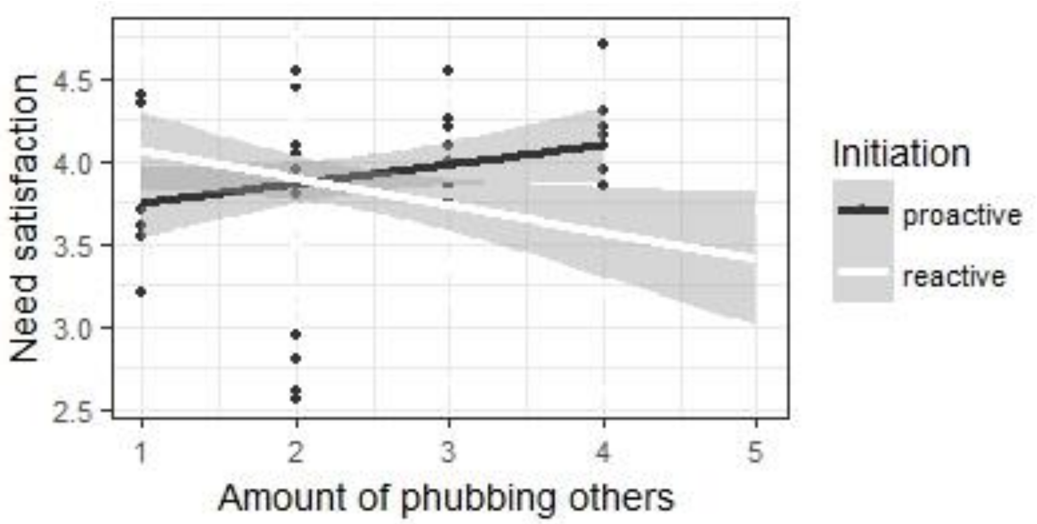

References

Aron, A., Aron, E. N., & Smollan, D. (1992). Inclusion of other in the self scale and the structure of interpersonal closeness. *Journal of Personality and Social Psychology, 63*(4), 596-612. doi: 10.1037/0022-3514.63.4.596

Kooiman, B. J., Li, W., Wesolek, M., & Kim, H. (2015). Validation of the relatedness scale of the intrinsic motivation inventory through factor analysis. *International Journal of Multidisciplinary Research and Modern Education, 1,* 302-311.

McAuley, E., Duncan, T., & Tammen, V. V. (1989). Psychometric properties of the Intrinsic Motivation Inventory in a competitive sport setting: A confirmatory factor analysis. *Research Quarterly for Exercise and Sport, 60,* 48-58. doi: 10.1080/02701367.1989.10607413
